# Personalized decision-making for acute cholecystitis: Understanding surgeon judgment

**DOI:** 10.3389/fdgth.2022.845453

**Published:** 2022-09-15

**Authors:** Amanda C. Filiberto, Philip A. Efron, Amanda Frantz, Azra Bihorac, Gilbert R. Upchurch, Tyler J. Loftus

**Affiliations:** ^1^ Department of Surgery, University of Florida Health, Gainesville, FL, United States; ^2^ Department of Anesthesiology, University of Florida Health, Gainesville, FL, United States; ^3^ Department of Medicine, University of Florida Health, Gainesville, FL, United States; ^4^ Intelligent Critical Care Center, University of Florida Health, Gainesville, FL, United States

**Keywords:** cholecystitis, cholecystectomy, cholecystostomy, surgical decision making, risks, complications

## Abstract

**Background:**

There is sparse high-level evidence to guide treatment decisions for severe, acute cholecystitis (inflammation of the gallbladder). Therefore, treatment decisions depend heavily on individual surgeon judgment, which is highly variable and potentially amenable to personalized, data-driven decision support. We test the hypothesis that surgeons' treatment recommendations misalign with perceived risks and benefits for laparoscopic cholecystectomy (surgical removal) vs. percutaneous cholecystostomy (image-guided drainage).

**Methods:**

Surgery attendings, fellows, and residents applied individual judgement to standardized case scenarios in a live, web-based survey in estimating the quantitative risks and benefits of laparoscopic cholecystectomy vs. percutaneous cholecystostomy for both moderate and severe acute cholecystitis, as well as the likelihood that they would recommend cholecystectomy.

**Results:**

Surgeons predicted similar 30-day morbidity rates for laparoscopic cholecystectomy and percutaneous cholecystostomy. However, a greater proportion of surgeons predicted low (<50%) likelihood of full recovery following percutaneous cholecystostomy compared with cholecystectomy for both moderate (30% vs. 2%, *p *< 0.001) and severe (62% vs. 38%, *p *< 0.001) cholecystitis. Ninety-eight percent of all surgeons were likely or very likely to recommend cholecystectomy for moderate cholecystitis; only 32% recommended cholecystectomy for severe cholecystitis (*p *< 0.001). There were no significant differences in predicted postoperative morbidity when respondents were stratified by academic rank or self-reported ability to predict complications or make treatment recommendations.

**Conclusions:**

Surgeon recommendations for severe cholecystitis were discordant with perceived risks and benefits of treatment options. Surgeons predicted greater functional recovery after cholecystectomy but less than one-third recommended cholecystectomy. These findings suggest opportunities to augment surgical decision-making with personalized, data-driven decision support.

## Introduction

Surgeons make complex, high-stakes, personalized recommendations when managing patients with acute cholecystitis. Evidence supports the safety and efficacy of laparoscopic cholecystectomy for patients with moderate cholecystitis characterized by significant leukocytosis, prolonged duration of symptoms, or signs of marked local inflammation like gallbladder gangrene, emphysema, or perforation (1–3). For patients with severe acute cholecystitis, (i.e., cholecystitis associated with organ dysfunction manifested as altered sensorium, cardiovascular instability, hypoxemia, acute kidney injury, cholestatic coagulopathy, or thrombocytopenia), high-level evidence is lacking, and guidelines are ambiguous regarding early cholecystectomy (surgical removal) vs. percutaneous cholecystostomy (image-guided drainage) (1, 2, 4–6). Therefore, in the absence of prohibitive risk for procedural interventions or patient preference for medical management alone, surgeons must recommend cholecystectomy or cholecystostomy using clinical judgment alone.

Unfortunately, individual surgeon judgment is highly variable and occasionally errant. In a prospective audit of complications among 4,743 surgical patients, diagnostic and judgment errors were the second-most common cause of preventable harm (7). In a survey of 7,905 members of the American College of Surgeons (ACS), surgeons reported that lapses in judgment were the most common cause of major errors, accounting for 32% (8). Surgical decision-making is often compromised by time constraints, uncertainty, and the lack of high-level evidence as well as concrete guidelines and recommendations from professional societies. Under these circumstances, surgeons may rely on heuristics or cognitive shortcuts, which can lead to bias and error, and can ignore important elements of personalized decision-making (9, 10). Variability in decision-making regarding operative and non-operative management has been described for several acute surgical conditions but not for cholecystitis, a condition for which evidence gaps force reliance on individual judgement (11).

To create a framework for understanding how and why surgeons decide between cholecystectomy and cholecystostomy, surgery attendings, fellows, and residents were surveyed regarding their estimated risks and benefits of laparoscopic cholecystectomy and percutaneous cholecystostomy for moderate and severe acute cholecystitis, as well as the likelihood that they would recommend cholecystectomy. Our null hypothesis was that there would be no difference in perceived risks and benefits between cholecystectomy and cholecystostomy, and that the likelihood of recommending cholecystectomy would be evenly distributed along a continuum ranging from very unlikely to very likely.

## Material and methods

### Study design

To identify patient and surgeon characteristics that are most influential in surgical-decision making for acute cholecystitis, a live, web-based survey was performed during Surgery Grand Rounds at a tertiary care academic center. After excluding four respondents that provided incomplete data, there were 50 respondents, including 27 attending surgeons and 23 surgery residents and fellows, representing a diverse set of training backgrounds and subspecialties. These subjects scanned a QR code or entered a web address and PIN number on their mobile device to access a VoxVote (Breda, Netherlands) live voting exercise. Participation was voluntary and anonymous. Respondents acknowledged that participation included consent for the reproduction and publication of data produced during the exercise. Institutional Review Board approval was obtained.

The survey questions are listed in Supplementary Table S1 and case scenarios are listed in Table 1. Briefly, surgeons reported their professional rank and rated their ability to accurately predict the risk of postoperative complications, their ability to make effective recommendations for operative management, and their surgical technical skills as being in the top half or bottom half of their peer group, defined as individuals with the same professional rank.

**Table 1:** Clinical scenarios.

**Scenario 1:** A 58-year-old male presents to the emergency department with a two-day history of right upper quadrant pain, nausea, and vomiting. Right upper quadrant ultrasound demonstrates cholelithiasis, gallbladder wall thickening, pericholecystic fluid, positive sonographic Murphy's sign, and common bile duct diameter 4 mm.
Past medical history: hypertension, hyperlipidemia, diabetes, obesity (body mass index 32 kg/m^2^).
Past surgical history: none.
Social history: quit smoking three years ago.
Medications: hydrochlorothiazide, atorvastatin, metformin.
Vital signs: heart rate = 98, blood pressure = 154/93 mmHg, respiratory rate = 20, oxygen saturation = 98%.
Laboratory values: serum bicarbonate = 22 mmol/L, serum creatinine =1.2 mg/dl, albumin = 3.6 g/dl, total bilirubin= 1.0 mg/dl, direct bilirubin = 0.5 mg/dl, white blood cell count = 19.7 × 10^9^/L, hemoglobin = 13.0 g/dl, hematocrit = 41%, platelet count = 211 × 10^9^/L, international normalized ratio = 0.9, glycated hemoglobin = 7.5%, lipase = 22 U/L.
1) Scenario 1: What is the probability of a serious complication within 30 days of laparoscopic cholecystectomy? a. 0%–2% b. 2%–5% c. 5%–10% d. 10%–20% e. >20% 2) Scenario 1: What is the probability of recovery within 30 days of laparoscopic cholecystectomy? a. <50% b. 50%–75% c. 75%–90% d. 90%–95% e. 95%–100% 3) Scenario 1: What is the probability of a serious complication within 30 days of percutaneous cholecystostomy? a. 0%–2% b. 2%–5% c. 5%–10% d. 10%–20% e. >20% 4) Scenario 1: What is the probability of recovery within 30 days of percutaneous cholecystostomy? a. <50% b. 50%–75% c. 75%–90% d. 90%–95% e. 95%–100% 5) Scenario 1: How likely are you to recommend laparoscopic cholecystectomy? a. Very unlikely b. Unlikely c. Neutral d. Likely e. Very Likely
**Scenario 2:** A 58-year-old male presents to the emergency department with a five-day history of right upper quadrant pain, nausea, vomiting, and delirium. Right upper quadrant ultrasound demonstrates cholelithiasis, gallbladder wall thickening and perforation, pericholecystic fluid, positive sonographic Murphy's sign, and common bile duct diameter 4 mm.
Past medical history: hypertension, hyperlipidemia, diabetes, obesity (body mass index 32 kg/m^2^)
Past surgical history: none
Social history: quit smoking three years ago
Medications: hydrochlorothiazide, atorvastatin, metformin
Vital signs: heart rate = 113, blood pressure = 103/78 mmHg, respiratory rate = 28, oxygen saturation = 92%
Laboratory values: serum bicarbonate = 19 mmol/L, serum creatinine =2.1 mg/dl, albumin = 2.9 g/dl, total bilirubin = 1.3 mg/dl, direct bilirubin = 0.5 mg/dl, white blood cell count = 21.3 × 10^9^/L, hemoglobin = 9.0 g/dl, hematocrit = 30%, platelet count = 92 × 10^9^/L, international normalized ratio = 1.4, glycated hemoglobin = 7.5%, lipase = 22 U/L
6) Scenario 2: What is the probability of a serious complication within 30 days of laparoscopic cholecystectomy? a. 0%–2% b. 2%–5% c. 5%–10% d. 10%–20% e. >20%
7) Scenario 2: What is the probability of recovery within 30 days of laparoscopic cholecystectomy? a. <50% b. 50%–75% c. 75%–90% d. 90%–95% e. 95%–100%
8) Scenario 2: What is the probability of a serious complication within 30 days of percutaneous cholecystostomy? a. 0%–2% b. 2%–5% c. 5%–10% d. 10%–20% e. >20%
9) Scenario 2: What is the probability of recovery within 30 days of percutaneous cholecystostomy? a. <50% b. 50%–75% c. 75%–90% d. 90%–95% e. 95%–100%
10) Scenario 2: How likely are you to recommend laparoscopic cholecystectomy? a. Very unlikely b. Unlikely c. Neutral d. Likely b. Very Likely

Serious complications include cardiac arrest, MI, pneumonia, progressive renal insufficiency, acute renal failure; PE, deep vein thrombosis, sepsis, respiratory failure; UTI, return to the OR, deep or organ space SSI, and wound disruption. Recovery is defined as being free of the immediate threats of the disease process and back to a reasonable level of baseline health.

A scenario of moderate acute cholecystitis was then presented, as described in the supplement. Moderate cholecystitis was defined by Tokyo Guidelines (1, 2). Respondents estimated the probability of a serious complication within 30 days of laparoscopic cholecystectomy, including cardiac arrest, myocardial infarction, pneumonia, progressive renal insufficiency, acute renal failure, pulmonary embolism, deep vein thrombosis, sepsis, respiratory failure, urinary tract infection, return to the operating room, deep or organ space surgical site infection, and wound disruption, in accordance with the ACS National Surgical Quality Improvement Program (NSQIP) definitions (11). Estimated risk for complications was classified as 0%–2%, 2%–5%, 5%–10%, 10%–20%, or >20%. Respondents then estimated the probability of recovery within 30 days of laparoscopic cholecystectomy, defined as being free of the immediate threats of the disease process and back to a reasonable level of baseline health (11). Estimated likelihood of recovery was classified as <50%, 50%–75%, 75%–90%, 90%–95%, or 95%–100%. The ACS NSQIP Surgical Risk Calculator was used to estimate the risk of serious complications within 30 days of laparoscopic cholecystectomy. The same two questions regarding complications and recovery were asked regarding percutaneous cholecystostomy for the same moderate cholecystitis scenario. Respondents were then asked to describe the likelihood that they would recommend cholecystectomy, classified as very unlikely, unlikely, neutral, likely, or very likely.

A second clinical scenario of severe acute cholecystitis was then presented, and the same five questions regarding complications and recovery for laparoscopic cholecystectomy and percutaneous cholecystostomy and the likelihood of recommending cholecystectomy were repeated. Severe cholecystitis was defined by Tokyo Guidelines (1, 2).

### Statistical analysis

Statistical analysis was performed in SPSS version 25 (IBM, Armonk, NY) with significance set at *α *= 0.05. Estimated risk of serious complication and recovery within 30 days following laparoscopic cholecystectomy vs. percutaneous cholecystostomy were compared by the Wilcoxon matched pairs signed rank test. The proportion of respondents recommending cholecystectomy for moderate vs. severe acute cholecystitis was compared by Fisher's Exact test. Univariable logistic regression was used to assess associations between the likelihood of recommending cholecystectomy and multiple potentially influential factors including professional rank, self-assessment of decision-making and operative skills, and estimations of risks and benefits of cholecystectomy and cholecystostomy. Based on results of the regression analysis, secondary analyses were performed comparing estimated risk of 30-day morbidity following laparoscopic cholecystectomy stratified by professional rank and self-identification as being in the top half of one's peer group in predicting complications, making treatment recommendations, and technical skills. These subgroups were compared by Fisher's exact test. The exploratory nature of this study and the absence of effect size estimates from prior studies precluded the performance of a power analysis.

## Results

### Predicted morbidity following cholecystectomy and cholecystostomy

**Figure 1:**
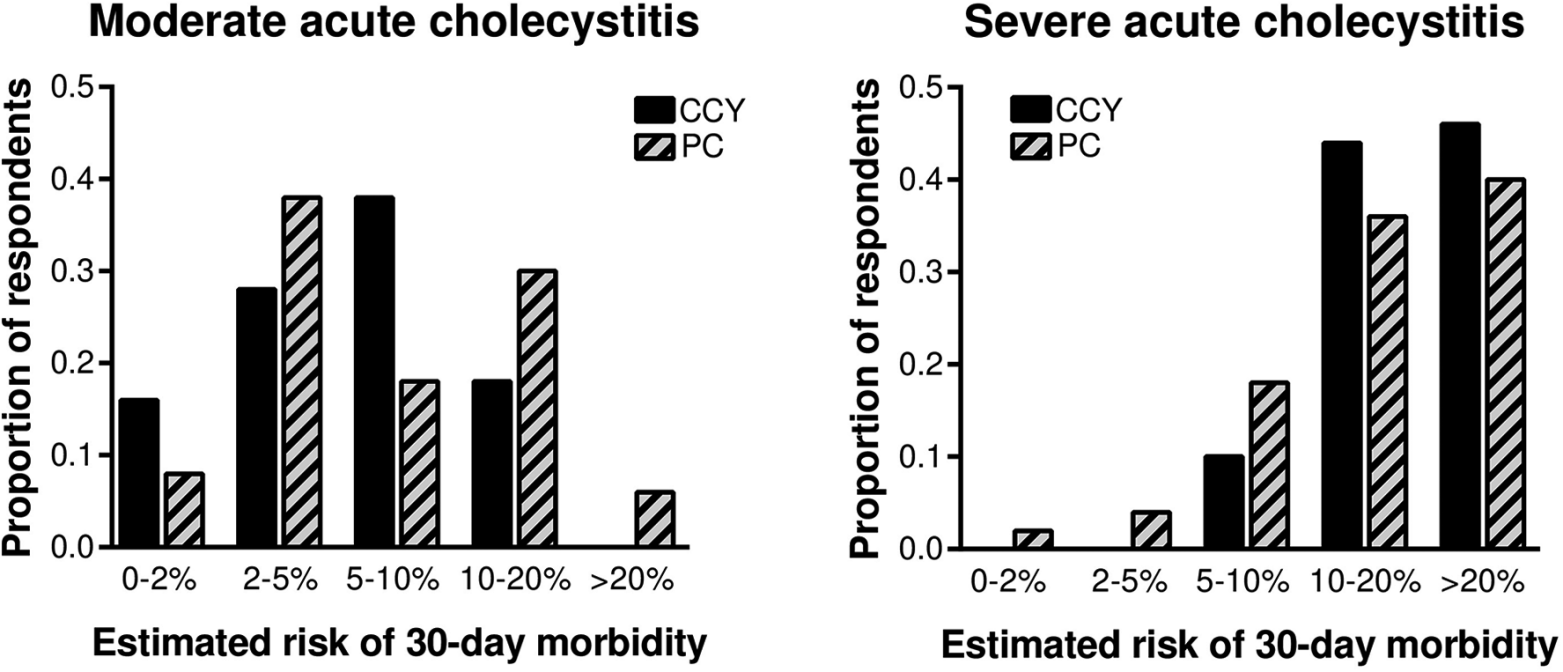
Surgeon-estimated 30-day morbidity was similar for laparoscopic cholecystectomy and percutaneous cholecystostomy for both moderate and severe cholecystitis. CCY, laparoscopic cholecystectomy; PC, percutaneous cholecystostomy.

Estimated 30-day morbidity was similar for laparoscopic cholecystectomy and percutaneous cholecystostomy for both moderate and severe acute cholecystitis (Figure 1). For the moderate cholecystitis scenario, the most common morbidity estimate for laparoscopic cholecystectomy was 5%–10%, selected by 38% (19/50) of all respondents; for percutaneous cholecystostomy, the most common morbidity estimate was 2–5%, selected by 38% (19/50). The ACS NSQIP Surgical Risk Calculator predicted a 5.6% chance of serious complications within 30 days of laparoscopic cholecystectomy. For the severe cholecystitis scenario, the most common morbidity estimate for laparoscopic cholecystectomy was >20%, selected by 46% (23/50); for percutaneous cholecystostomy the most common morbidity estimate was also >20%, selected by 40% (20/50). The ACS NSQIP Surgical Risk Calculator predicted a 9.2% chance of serious complications within 30 days of laparoscopic cholecystectomy.

### Predicted recovery following cholecystectomy and cholecystostomy

**Figure 2:**
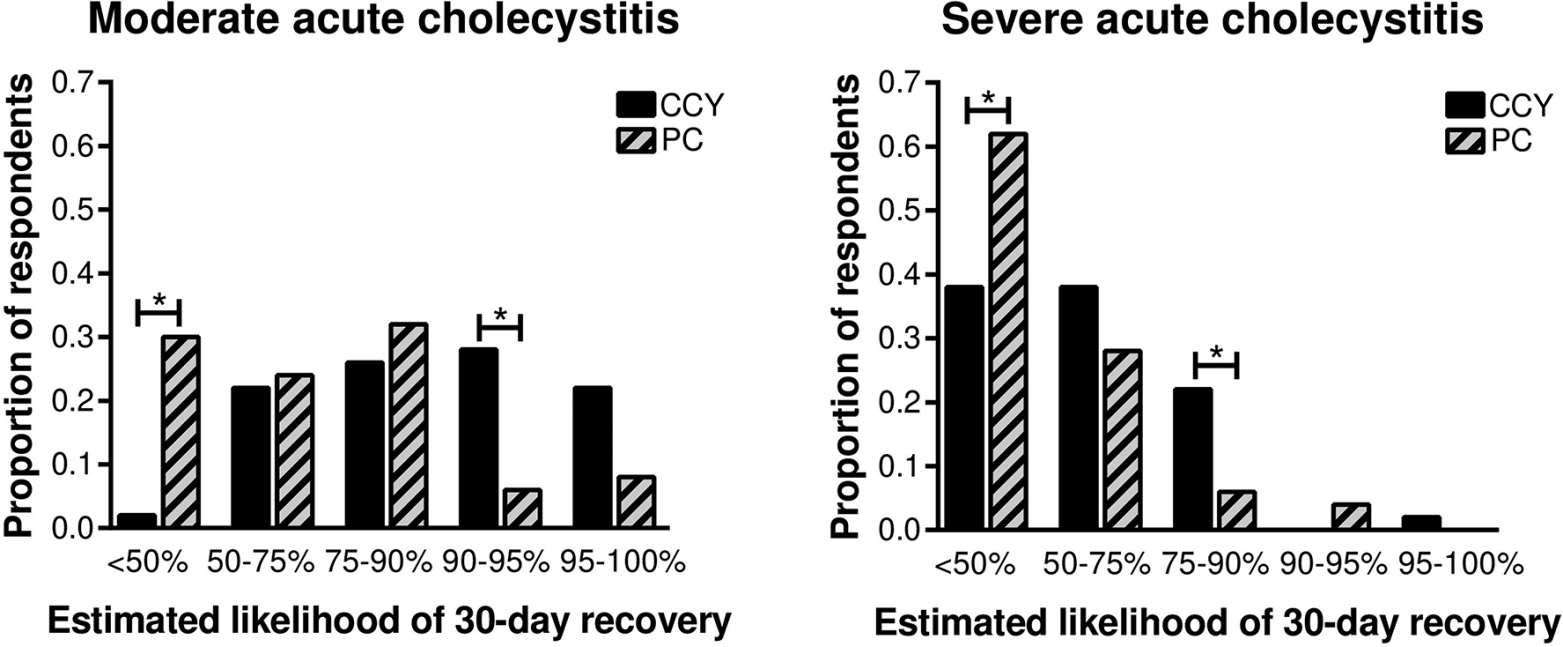
Surgeon-estimated 30-day recovery favored laparoscopic cholecystectomy over percutaneous cholecystectomy for both moderate and severe cholecystitis. CCY, laparoscopic cholecystectomy; PC, percutaneous cholecystostomy. *p < 0.05 between groups.

Estimated 30-day recovery favored laparoscopic cholecystectomy over percutaneous cholecystectomy for both moderate and severe acute cholecystitis (Figure 2). For the moderate cholecystitis scenario, only one respondent (2%) predicted a <50% chance of recovery following laparoscopic cholecystectomy, whereas 30% (15/50) predicted a <50% chance of recovery following percutaneous cholecystostomy (*p *< 0.001). Similarly, for the severe cholecystitis scenario, 38% (19/50) predicted a <50% chance of recovery following laparoscopic cholecystectomy, whereas 62% (31/50) predicted a <50% chance of recovery following percutaneous cholecystostomy (*p *< 0.001).

### Likelihood of recommending cholecystectomy

**Figure 3:**
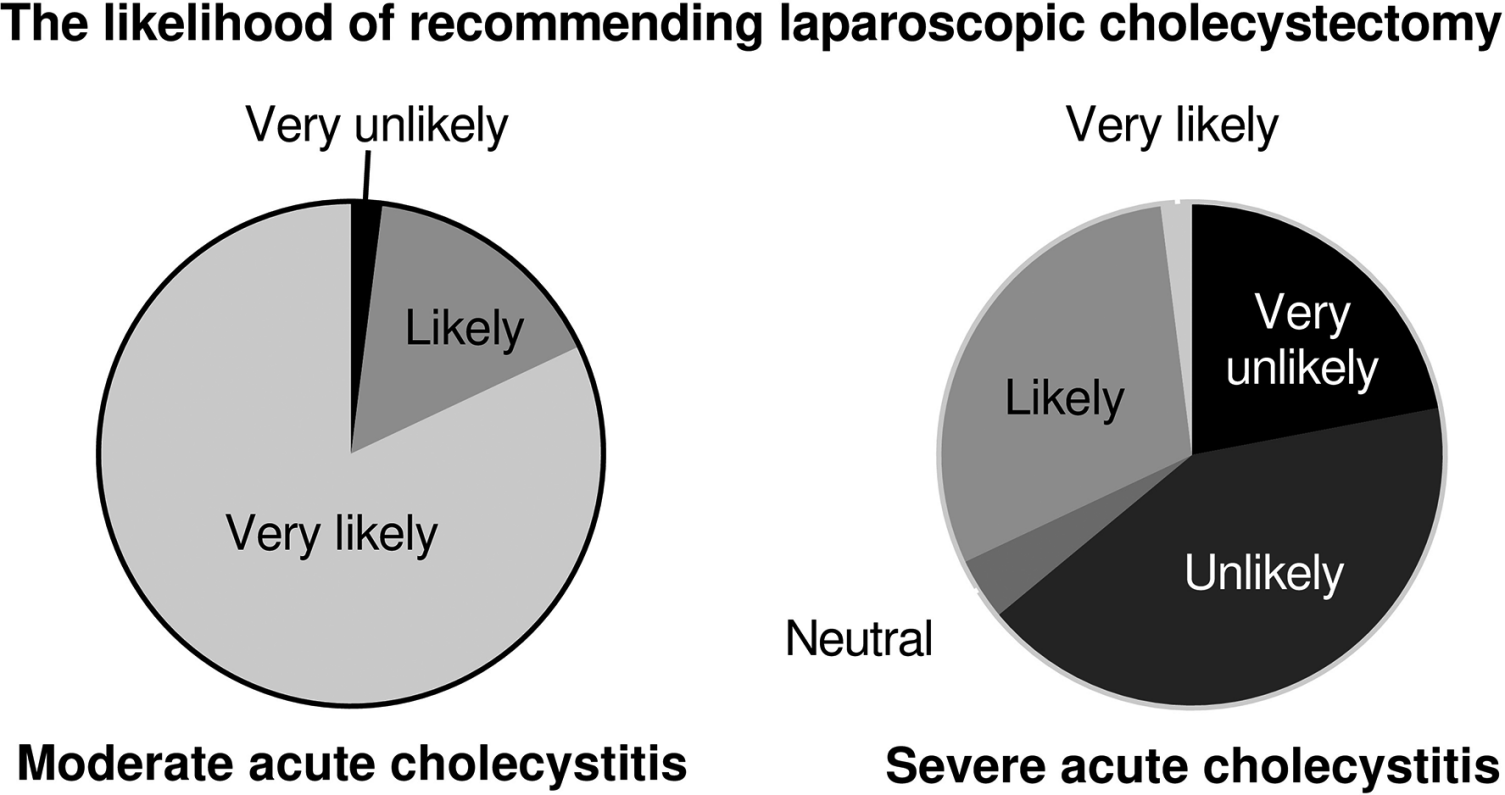
Surgeons were more likely to recommend laparoscopic cholecystectomy for patients with moderate acute cholecystitis compared with severe cholecystitis (98% vs. 32%, *p *< 0.001).

For the moderate acute cholecystitis scenario, 98% (49/50) of all respondents were likely or very likely to recommend cholecystectomy. For the severe cholecystitis scenario, only 32% (16/50) were likely or very likely to recommend cholecystectomy (Figure 3, *p *< 0.001). Univariable logistic regression demonstrated that the only factor associated with the likelihood of recommending laparoscopic cholecystectomy for severe cholecystitis was estimated 30-day morbidity (Table 2).

**Table 2:** Univariable predictors recommending laparoscopic cholecystectomy for severe acute cholecystitis.

Factor	Odds ratio	95% CI	*p*
Attending surgeon^a^ (vs. resident or fellow)	2.48	0.71–8.67	0.156
Self-identifying as top half in predicting complications relative to peers^b^	1.86	0.49–7.00	0.360
Self-identifying as top half in making treatment recommendations relative to peers^b^	4.33	0.85–22.23	0.079
Self-identifying as top half in technical skills relative to peers^b^	2.36	0.56–9.97	0.241
Estimated 30-day morbidity^c^ following: Laparoscopic cholecystectomy			
0%–2%	–	–	–
2%–5%	–	–	–
5%–10%	11.00	1.12–108.50	0.040
10%–20%	1.43	0.43–4.72	0.558
>20%	0.26	0.07–0.98	0.047
Percutaneous cholecystostomy			
0%–2%	–	–	–
2%–5%	–	–	–
5%–10%	0.55	0.10–3.01	0.492
10%–20%	1.63	0.48–5.52	0.435
>20%	0.58	0.16–2.02	0.389
Estimated 30-day recovery^d^ following: Laparoscopic cholecystectomy			
<50%	0.65	0.18–2.29	0.501
50%–75%	0.65	0.18–2.29	0.501
75%–90%	2.12	0.54–8.40	0.284
90%–95%	–	–	–
95%–100%	–	–	–
Percutaneous cholecystostomy			
<50%	0.32	0.09–1.11	0.073
50%–75%	3.00	0.83–10.91	0.095
75%–90%	1.07	0.09–12.71	0.959
90%–95%	2.20	0.13–37.59	0.586
95%–100%	–	–	–

CI, confidence interval.

^a^ Professional rank, relative to surgery residents and fellows.

^b^ Respondents rated their abilities relative to peers, defined as individuals with the same professional rank.

^c^ Cardiac arrest, MI, pneumonia, progressive renal insufficiency, acute renal failure; PE, deep vein thrombosis, sepsis, respiratory failure; UTI, return to the OR, deep or organ space SSI, and wound disruption.

**Figure 4:**
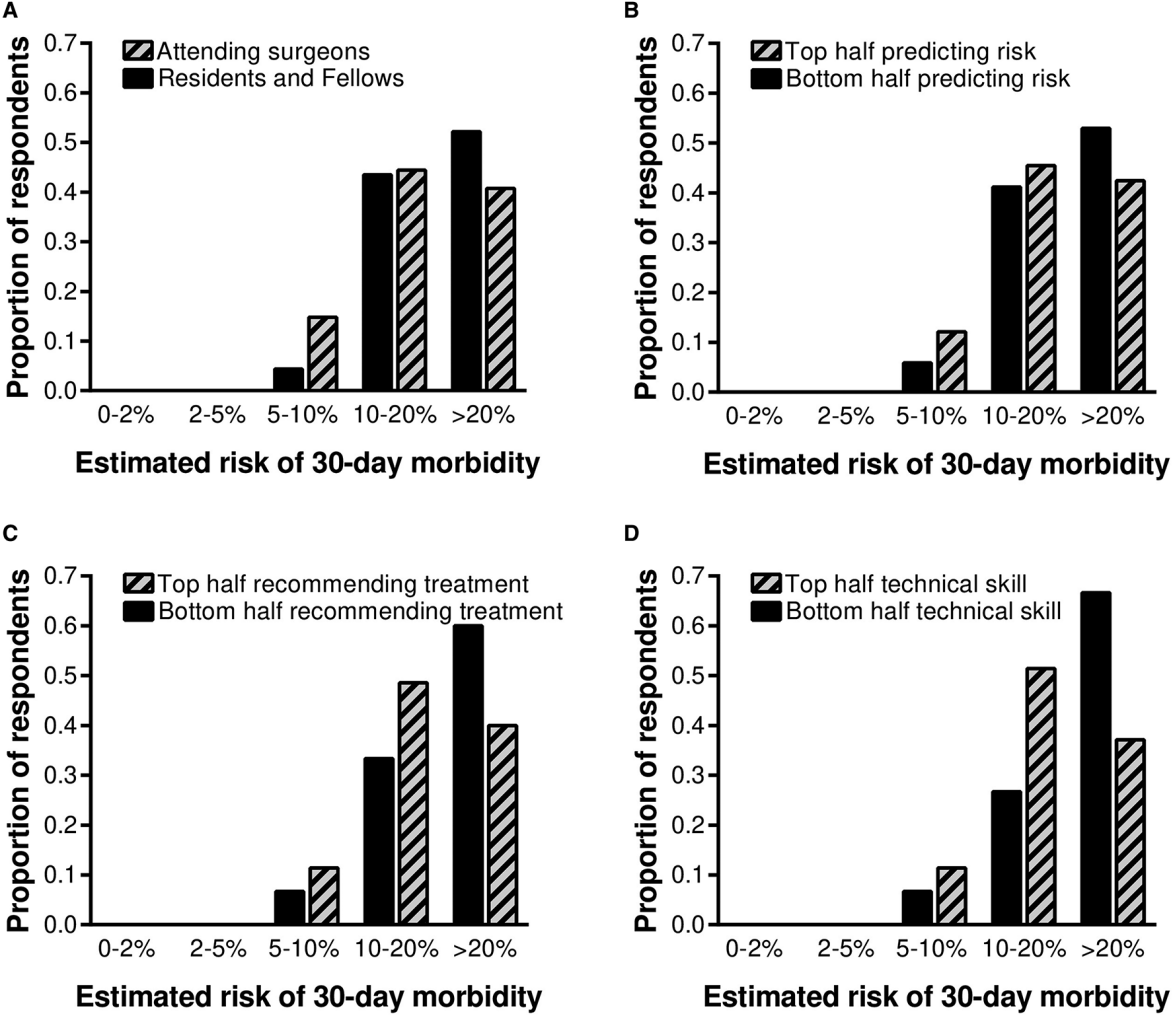
Predicted postoperative morbidity was stratified by professional rank and self-identification as being in the top half of one's peer group in predicting complications, making treatment recommendations, and technical skills. Thirty-seven percent of all surgeons who self-identified as top half for technical skills predicted a high (>20%) chance of serious complications following laparoscopic cholecystectomy, compared with 67% of all surgeons who self-identified as bottom half for technical skills (*p *= 0.070).

Based on this finding, a secondary analysis was performed to determine whether professional rank or self-identifying as being in the top half of one's peer group in predicting complications, making treatment recommendations, and technical skills were associated with estimated 30-day morbidity. There were no significant differences in predicted postoperative morbidity comparing trainees with attending surgeons or when respondents were stratified according to self-reported ability to predict complications or make treatment recommendations (Figure 4). Surgeons who self-identified as being in the top half of their peer group for technical skills were somewhat less likely to predict a high (>20%) likelihood of serious complications following laparoscopic cholecystectomy, though the difference was not statistically significant (37% vs. 67%, *p *= 0.070).

## Discussion

Treatment recommendations for a patient with severe cholecystitis were discordant with perceived risks and benefits of treatment options. Surgeons estimated that cholecystectomy and cholecystostomy would have similar 30-day morbidity, but that patients were more likely to recover to their baseline level of health if they underwent cholecystectomy. However, less than one third of all surgeons recommended cholecystectomy for severe cholecystitis. When univariable regression was performed to determine which factors predicted treatment recommendations, 30-day morbidity following cholecystectomy was the only significant predictor. These findings suggest that surgeons recommended non-operative management to avoid surgical complications, even though they believed that surgery was best for the patient, representing a deviation from personalized, patient-centered decision-making.

Several factors may contribute to this phenomenon. Major complications are associated with emotional exhaustion, depression, and burnout among surgeons (8, 12). A complication that is memorable for its severity or consequences may disproportionately affect future decisions, making a surgeon less likely to operate on a patient who would benefit from an operation (9, 10). Complications may tarnish a surgeon's reputation and decrease referrals, especially for female surgeons (13, 14). Surgeons may also practice defensive medicine, avoiding high-risk patients or procedures in order to avoid litigation and legal consequences, defending themselves rather than the patient (15, 16).

Additionally, in patients with severe acute cholecystitis, high-level evidence for laparoscopic cholecystectomy vs. percutaneous cholecystostomy are lacking as most studies are retrospective, have limited sample sizes, and have conflicting conclusions (3–6, 17–19). A recent meta-analysis including over 300,000 patients found that critically ill patients with acute cholecystitis who underwent cholecystectomy had improved mortality, length of hospital stay and rate of readmission for biliary complaints as compared with patients who underwent percutaneous cholecystostomy (20). This meta-analysis, however, did not include any randomized controlled trials, reflects a high degree of heterogeneity and were from single-centered studies. Findings from this meta-analysis were later validated by a randomized controlled trial including 142 patients, showing that laparoscopic cholecystectomy reduced the rate of major complications compared with percutaneous catheter drainage in high-risk patients with acute cholecystitis (21). Despite this data, surgeons may be reticent to embark on a high-risk course of surgical management based on one relatively small, albeit it well designed, study. Therefore, in the absence of prohibitive risk for procedural interventions or patient preference for medical management alone, surgeons may rely primarily on highly variable individual judgement in choosing between cholecystectomy or cholecystostomy.

Estimations of risks and benefits were highly variable, as were treatment recommendations for severe cholecystitis. Previous work has similarly demonstrated significant variability among surgeons in predicting risks and benefits and recommending surgery (11, 22–24). In a survey of members of the ACS, surgeons perceived operative and non-operative risks and benefits for acute surgical diseases to be highly variable, especially for scenarios in which evidence and guidelines are lacking or controversial (11). For acute appendicitis, more than 30% of all surgeons were highly likely to recommend appendectomy, and more than 20% were highly unlikely to recommend appendectomy. Less than half of all observed variability in the decision to operate could be explained by perceived risks and benefits of operative and non-operative management, supporting the hypothesis that other factors deter surgeons from patient-centered decision-making, as previously discussed. Still, variability in the decision to operate appears to be influenced heavily by perceived risks of different management strategies. In the present study, there was substantial disagreement between surgeons and ACS NSQIP Surgical Risk Calculator predictions, consistent with observed disagreement between surgeons and predictions generated by other risk calculators in previous studies; between surgeons and risk calculators, the calculators tend to have greater predictive performance (25, 26). These findings from other studies suggest potential utility to anchor surgeon judgement with accurate, objective risk assessments.

Surgeons predicted that laparoscopic cholecystectomy and percutaneous cholecystostomy would have similar 30-day morbidity, but that patients who underwent surgery would have a greater likelihood of recovery to baseline. Yet, less than one third of surgeons actually recommended surgery for a patient with severe acute cholecystitis; instead they recommended a non-operative strategy that was discordant with their belief that patients who undergo surgery recover faster. Surgeons often make complex, high-stakes decisions under time constraints and uncertainty, which can lead to suboptimal decisions and patient care. With the emerging availability of artificial intelligence prediction models, there is potential to transform surgical care by informing the decision to operate, providing patients and their caregivers with accurate prognostic information, and recommending optimal treatment strategies through machine learning techniques (27–29). Findings from our study suggest potentially adverse variability in surgical decision-making, and other studies suggest that well-designed AI-enabled decision support has the potential to standardize and decrease the variability of decision-making, though there is sparse high-level evidence to support this hypothesis (27–31).

These artificial intelligence techniques can aide surgeons in the complex task of risk stratification for intervention, using personalized data-driven support to make surgical decisions and optimize outcomes (30). For example, reinforcement learning can use large sets of complex patient-specific input data to identify actions yielding the greatest probability of achieving a goal following a sequence of events as uncertain conditions evolve (32). In theory, deep reinforcement learning could be used to incorporate an expanded set of input data (vital signs, lab values, imaging, etc.) to determine whether percutaneous cholecystostomy or laparoscopic cholecystectomy is more likely to yield optimal patient-centered outcomes, such as 30-day recovery, for a specific patient. The integration of surgeon judgment with patient outcome optimization such as reinforcement learning methods could help standardize care at an institutional level and ideally health systems of different scales.

This study included surgeons from a single institution, limiting the generalizability of these findings. In addition, varying levels of experience managing critically ill patients with severe cholecystitis among attending surgeons, fellows, and residents from different subspecialties introduces variability that may not be present in many practice settings. Based on the number of participants in the study, this study may have been underpowered to detect some potentially important and clinically significant differences, such as the observation that surgeons who self-identified as being in the top half of their peer group for technical skills were half as likely to predict a high likelihood of postoperative morbidity, which was not statistically significant in this study. The lack of prior evidence on this topic precludes the performance of a power analysis. Theoretically, surgeons from a single institution may be more likely to make uniform treatment recommendations consistent with an institutional culture, but this was not observed. Finally, this study demonstrated an association between perceived risk of surgical complications and treatment recommendations, but does not establish causality. Future research should seek to address this knowledge gap in a larger sample of surgeons from multiple institutions and practice environments, using the methods and analytic framework established by this work, ideally in a prospective, clinical setting. This can be used to better understand patient and surgeon characteristics that are most influential in a surgeon's decision to operate, and integrated with artificial intelligence models to make personalized, data-driven decision support in order to improve patient care.

## Conclusions

Surgeons predicted that laparoscopic cholecystectomy and percutaneous cholecystostomy would have similar 30-day morbidity, but greater likelihood of recovery to baseline health following cholecystectomy. Despite this, less than one third of all surgeons recommended cholecystectomy for a patient with severe acute cholecystitis. Treatment recommendations were discordant with perceived risks and benefits of treatment options; surgeons recommended a non-operative strategy that they thought was inferior, representing a deviation from personalized, patient-centered decision-making. Avoidance of surgical complications was the only factor that was associated with this discrepancy. These findings suggest opportunities to augment surgical decision-making with personalized, data-driven decision support.

## Data Availability

The original contributions presented in the study are included in the article/**Supplementary Material**, further inquiries can be directed to the corresponding author/s.
