# Supplementary material for: Personalized decision-making for acute cholecystitis: Understanding surgeon judgment
Source: Front Digit Health. 2022 Sep 15;4:845453. doi: 10.3389/fdgth.2022.845453 (PMC9632988; doi:10.3389/fdgth.2022.845453)
Supplement: Supplementary file 1 [file Table1.docx]

**Survey questions**

1. I understand that participation in this exercise includes consent for the reproduction and publication of data produced during this exercise.
   1. Yes
2. Please select your professional rank.
   1. Attending
   2. Fellow
   3. Resident
3. Please rate your ability to accurately predict the risk of postoperative complications relative to your peers.^a^
   1. Top half
   2. Bottom half
4. Please rate your ability to make effective recommendations for operative management relative to your peers.^a^
   1. Top half
   2. Bottom half
5. Please rate your surgical technical ability relative to your peers.^a^
   1. Top half
   2. Bottom half
